# Supplementary material for: Hybrid integrated ultra-low linewidth coil stabilized isolator-free widely tunable external cavity laser
Source: Nat Commun. 2025 Jul 1;16:5944. doi: 10.1038/s41467-025-61122-4 (PMC12216169; doi:10.1038/s41467-025-61122-4)
Supplement: Supplementary file 1 — Supplementary Information [file 41467_2025_61122_MOESM1_ESM.pdf]

Supplemental information

Hybrid integrated ultra-low linewidth coil stabilized isolator-free widely tunable external cavity laser

David A. S. Heim[1], Debapam Bose[1], Kaikai Liu[1], Andrei Isichenko[1], and Daniel J. Blumenthal[1*]
[1]Department of Electrical and Computer Engineering, University of California Santa Barbara, Santa Barbara, CA 93106 USA.
[*]Corresponding author (danb@ucsb.edu)

Supplementary Note 1: Introduction

This supplemental section provides additional information on the design and performance of the hybrid-integrated external cavity tunable laser (ECTL), including a diagram of the working principle of the dual-ring ECTL, measurement of the ring resonator Qs, measurements of the 10-meter coil resonator, more information regarding the free-running and integrated coil-stabilized frequency noise measurements, as well as a calculation of the optical feedback parameter.

The basic working principle of the dual-ring ECTL is illustrated in Supplementary Fig. 1a. The RSOA has a broad power spectrum and the two ring resonators, with slightly different radii, provide linewidth narrowing where the two resonances overlap. Changing the resonance of one of the rings adjusts which modes overlap and thereby tunes the laser up to one Vernier FSR. The back mirror of the RSOA and the loop mirror of the external Si_3N_4 circuit form the extended laser cavity and has associated Fabry Perot modes.

Supplementary Note 2: ECTL design

The layer stack for the low-loss Si_3N_4 waveguide platform is indicated in Supplementary Fig. 1b. The ECTL waveguide dimension is 2.6 µm by 80 nm resulting in a dilute optical mode to reduce scattering losses. Measurement of the quality factor (Q) of the intracavity rings, shown in Supplementary Fig. 1c, yield loaded and intrinsic-Qs of 0.6 and 3.5 million, respectively. The rings were intentionally heavily coupled to reduce cavity coupling losses to lower the lasing threshold at the expense of having higher loaded-Qs and longer photon lifetimes. This is a design parameter that can be optimized in future designs.

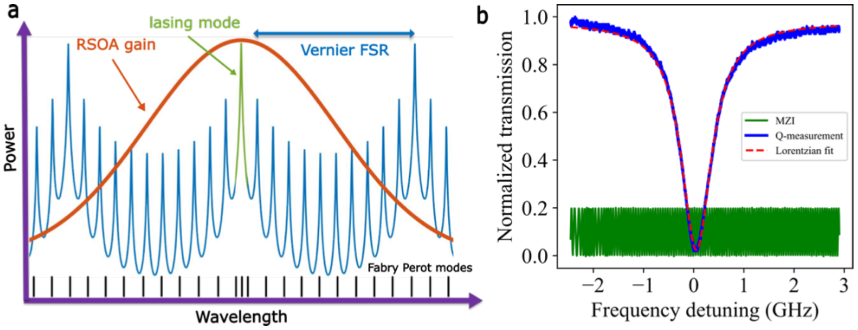

Supplementary Fig. 1 ECTL working principle and characterization. a An illustration of the basic working principle of the dual-ring external cavity tunable laser (ECTL). **b** Q-measurement of one of the ECTL rings (blue) fitted to a Lorentzian function (red) to extract Q values of 0.65 million loaded and 3.5 million intrinsic. An 18 MHz fiber-MZI (green) is used to calibrate the frequency detuning.

Supplementary Note 3: 10-meter-coil resonator design and characterization

The 10-meter coil resonator has a waveguide dimension of 6 μm by 80 nm utilizing the fundamental TE mode. The coil spiral center-to-center waveguide spacing is 25 μm, and the circular S-bend diameter in the center of the coil waveguides is ~3.6 mm, resulting in a much smaller device size and making it possible to wrap the 10-meter-coil waveguide on a single die size (21.6 mm by 26 mm). The bus-resonator directional coupler uses a 2.5 μm coupler gap and 1.5 mm coupling length. The bus waveguide is tapered from 6 μm to 1.5 μm for better fiber-to-chip edge coupling. The fiber-pigtailed device is packaged with a metal enclosure for better handling capabilities, shown in Supplementary Fig. 2a. The FSR is measured to be around 19.4 MHz, and the intrinsic Q reaches above 300 million around 1600 nm, shown in Supplementary Fig. 2d.

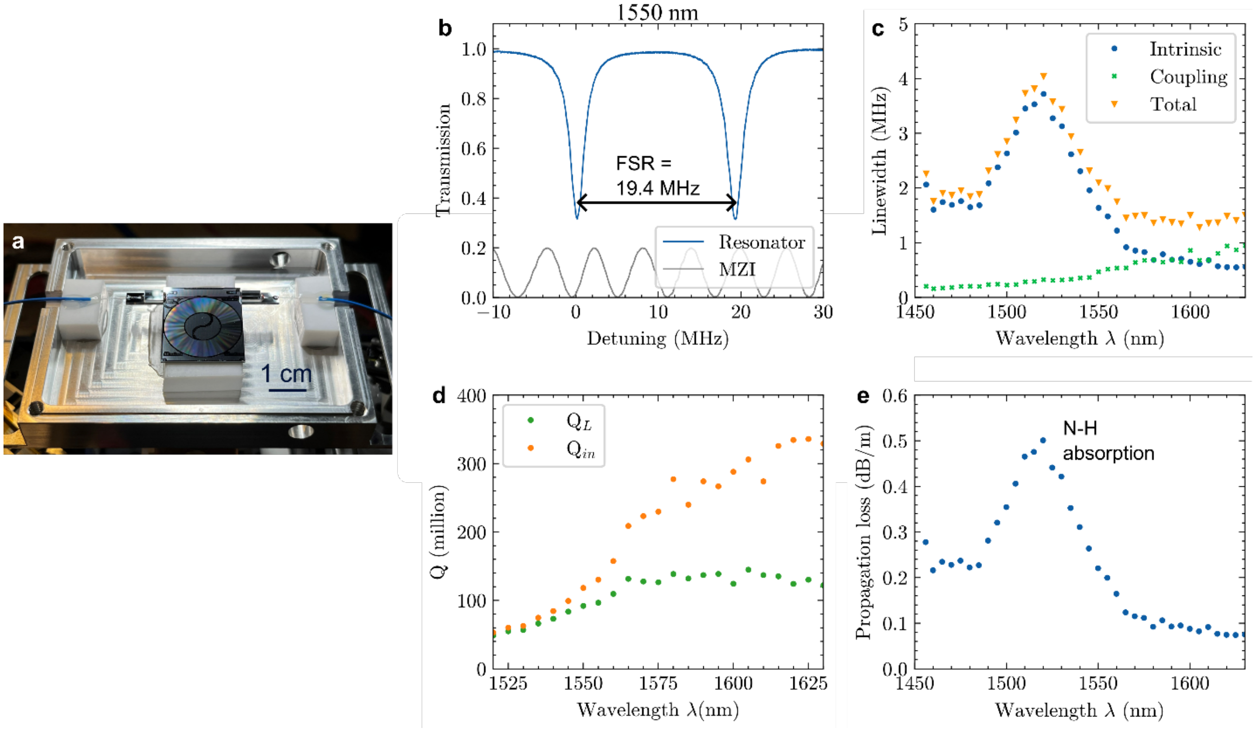

Supplementary Fig. 2. 10-meter-coil waveguide reference resonator design and testing. a 10-meter-coil resonator with PM-SMF28 fibers pig-tailed and packaged in a metal enclosure. **b** Spectral scan of the coil resonator resolves the resonance linewidth and FSR. **c** Intrinsic, coupling and total resonator linewidths in MHz are measured from 1450 nm to 1630 nm. **d** Intrinsic and loaded Qs are estimated from the resonator linewidth measurements. **e** Waveguide propagation loss is also estimated, showing an absorption peak around 1520 nm.

Supplementary Note 4: Frequency noise measurements

Plotted in Supplementary Fig. 3a is the FN of the free running ECTL (blue) measured using an unbalanced fiber-MZI as an optical frequency discriminator (OFD) and reading out the self-delayed homodyne signal on a balanced photodiode. The dashed lines in red, green, and orange indicate calculated estimates of the noise floors of the photodetector noise, thermal refractive noise

and photothermal noise of the ECTL intracavity ring resonators. The photo-thermal frequency noise spectrum induced by optical power fluctuations in the external cavity laser is described by,

$$S_{PTN}(f) = \left[\frac{\Delta f_{opt}}{P_{opt}}\right]^2 H_{th}^2(f) P_{opt}^2 S_{RIN} \quad (1)$$

where S_{RIN} is the experimentally measured relative intensity noise (RIN) spectrum (Supplementary Fig. 4c), P_{opt} is the estimated on-chip optical power, $H_{th}(f)$ is the thermal frequency response which can be estimated from COMSOL simulations, and $\frac{\Delta f_{opt}}{P_{opt}}$ describes the photo-thermal red shift strength that includes an estimation of ξ the absorption loss fraction which can be estimated experimentally. Our previous papers provide extensive details on this calculation, for example ref [1].

When we stabilize the ECTL to the 10-meter coil reference cavity the FN at certain low frequency offsets drops by five orders of magnitude, in which case noise due to the fiber-MZI can dominate over the laser noise. To resolve this, we make additional close-to-carrier (CTC) measurements for the locked ECTL by mixing it with a stable reference laser (SRL) system and measuring the heterodyne beatnote signal on a frequency counter. The SRL system includes a Vescent fiber frequency comb locked to a single frequency Rock fiber laser that is itself locked to an ultralow expansion (ULE) cavity. The CTC measurement of the stabilized-ECTL is plotted in Supplementary Fig. 3b (orange) and becomes limited by the speed of the frequency counter at frequency offsets above ~1 kHz. The OFD measurement of the locked-ECTL is plotted (green), and the two can be stitched together (vertical black line) to give a more accurate measurement of the laser FN and to calculate the integral linewidth. See refs [1–5] for more information.

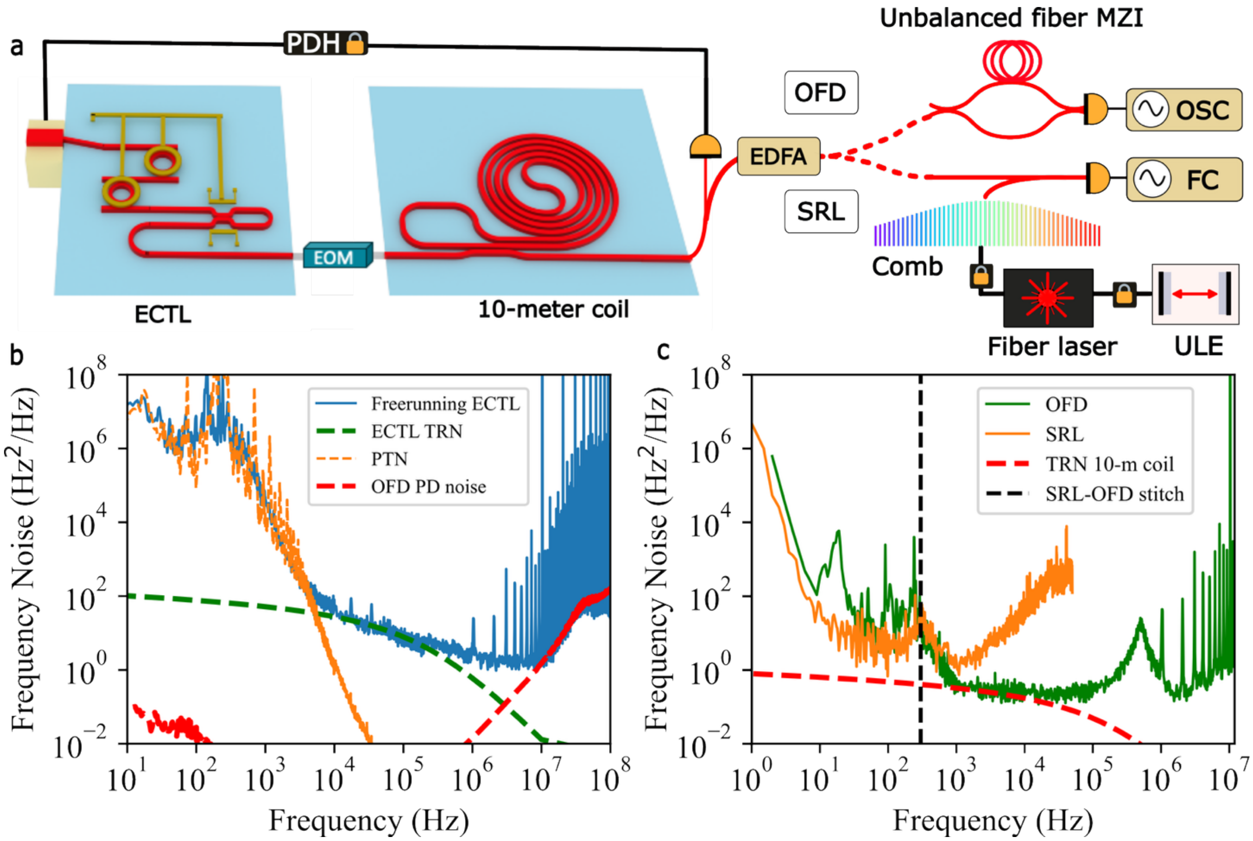

Supplementary Fig. 3 ECTL frequency noise measurements. a The frequency noise (FN) of the coil-locked ECTL is measured using two independent techniques: for FN below 3 kHz the ECTL output is mixed with a stable reference laser (SRL) that consists of an optical frequency comb locked to a single frequency Rock fiber laser that in turn is stabilized to an ultralow expansion (ULE) reference cavity, and the heterodyne beatnote signal is measured on a frequency counter (FC). For FN above 3 kHz an unbalanced fiber-MZI is used as an optical frequency discriminator (OFD) and the self-delayed homodyne signal is measured on a balanced photodiode and an oscilloscope (OSC). **b** Frequency noise power spectrum of the freerunning ECTL (blue) at 1550 nm measured using an MZI as an optical frequency discriminator. The orange, red, and green dashed curves are calculated estimates of the thermal refractive and photothermal noise floor of the two intracavity rings and the OFD photodetector noise, respectively. **c** FN of the coil-locked laser measured using two methods: OFD (green) and SRL (orange). The black vertical lines indicates where the two are stitched together for the composite locked-ECTL measurements.

The ECTL operates across 60 nm tuning. FN measurements of the freerunning and locked ECTL across the tuning range are plotted in Supplementary Fig. 4a,b showing fundamental linewidths between 3-7 Hz and 1/π-integral linewidths from 27 to 60 Hz. Relative intensity noise (RIN) measurements across the ECTL tuning range are also included in Supplementary Fig. 4c.

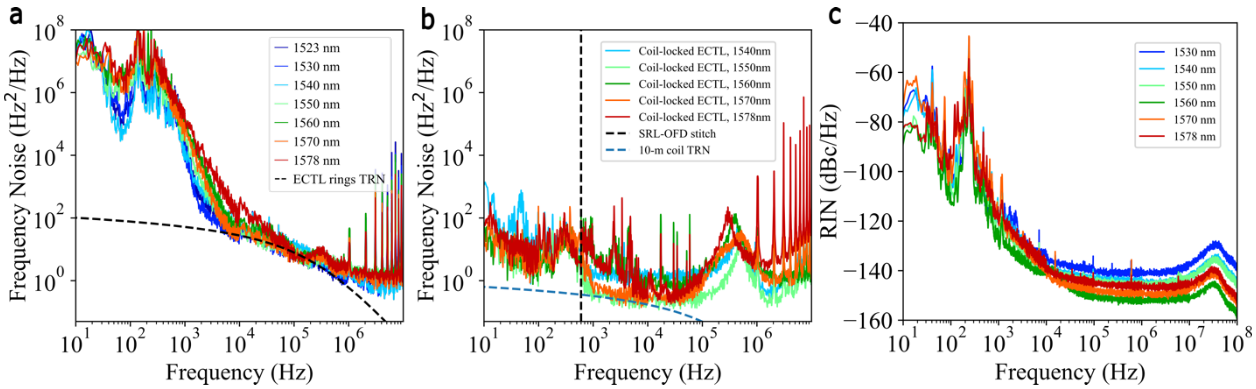

Supplementary Fig. 4 Additional free running and coil-locked ECTL frequency noise measurements. a FN measurements of the freerunning ECTL at various points across the tuning range plotted with the calculated TRN limit of the ECTL rings (black-dashed). **b** FN of the ECTL stabilized to the 10-m coil reference cavity at various points across the tuning range using two independent measurement techniques (stitched together at the black vertical line) and plotted with the calculated TRN limit of the 10-m coil (blue-dashed). **c** Measurement of relative intensity noise (RIN) of the ECTL at various points across the tuning range.

Supplementary Note 5: Optical feedback

Optical feedback in a semiconductor laser can be quantified by the feedback parameter C [6–8],

$$C = \frac{\tau_{ext} r_{ext}}{\tau_{laser} r_{laser}} (1 - |r_{laser}|^2)\sqrt{1+\alpha^2} \tag{1}$$

where r_{ext} and r_{laser} are the reflectivity of the external reflection and the laser mirror, τ_{ext} and τ_{laser} are the external cavity and laser cavity round trip lifetimes, and α is the linewidth enhancement factor. We can then compare the relative isolation of two lasers by taking the ratio of their C-parameters where the ratio is only dependent on characteristics of the laser: τ, r and α.

$$\frac{C_1}{C_2} = \frac{\tau_{laser_2}}{\tau_{laser_1}} \frac{r_{laser_2}}{r_{laser_1}} \frac{1-|r_{laser_1}|^2}{1-|r_{laser_2}|^2} \frac{\sqrt{1+\alpha_1^2}}{\sqrt{1+\alpha_2^2}} \tag{2}$$

The robustness of our ECTL to feedback light arises predominantly from two attributes. The first, is the extended cavity photon lifetime, τ_{laser}, due to the two high-Q intracavity rings. We can approximate the overall roundtrip photon lifetime of the dual-ring ECTL as a sum of the Fabry-Perot (FP) and individual ring resonator cavity lifetimes[9]. The FP cavity is formed by the back mirror of the RSOA (R_1) and the Si_3N_4 waveguide Sagnac loop mirror (R_2), and subject to internal losses (η), such as the coupling loss at the interface between the gain chip and the PIC, and has an associated lifetime of:

$$\tau_{FP} = \frac{-2L}{c} ln(R_1 R_2 (1-\eta)^2)^{-1} \tag{3}$$

The rings each contribute an additional $\tau_{RR} = \frac{\lambda Q}{2\pi c}$ and one full roundtrip requires four passes through a ring: $\tau_{ectl} = \tau_{FP} + 4\tau_{RR}$. For the ECTL we estimate that τ_{ectl} = 2.3 ns. The second attribute that contributes to the robustness to optical feedback is the relatively high reflectivity of the Sagnac loop mirror, which for our design is ~75%. The low loss intracavity rings store enough power within the cavity to support a high mirror reflectivity and still provide useful output power from the laser. Compared to a conventional III-V DFB laser that has an internal Q of ~1×10^4 and

front mirror reflectivity of <1 %, we estimate from equation (2) that the ECTL has inherent isolation of approximately 45 dB. Experimentally we demonstrate no degradation in the frequency noise of the ECTL under optical feedback of up to -10 dB. Compared to a commercial III-V DFB that undergoes coherence collapse at -40 dB of feedback, this demonstrates a 30 dB improvement in robustness to feedback.

Supplementary References

1. Liu, K. *et al.* Photonic circuits for laser stabilization with integrated ultra-high Q and Brillouin laser resonators. *APL Photonics* **7**, 096104 (2022).
2. Liu, K. *et al.* 36 Hz integral linewidth laser based on a photonic integrated 4.0 m coil resonator. *Optica* **9**, 770 (2022).
3. Liu, K. *et al.* Common cavity waveguide coil-resonator stabilized hybrid integrated WDM laser with 89 Hz integral linewidth. in *2024 Optical Fiber Communications Conference and Exhibition (OFC)* 1–3 (2024).
4. Gundavarapu, S. *et al.* Sub-hertz fundamental linewidth photonic integrated Brillouin laser. *Nature Photon* **13**, 60–67 (2019).
5. Chauhan, N. *et al.* Visible light photonic integrated Brillouin laser. *Nat Commun* **12**, 4685 (2021).
6. Zhang, Z. *et al.* High-Speed Coherent Optical Communication With Isolator-Free Heterogeneous Si/III-V Lasers. *Journal of Lightwave Technology* **38**, 6584–6590 (2020).
7. Gomez, S. *et al.* High coherence collapse of a hybrid III–V/Si semiconductor laser with a large quality factor. *J. Phys. Photonics* **2**, 025005 (2020).
8. Harfouche, M. *et al.* Kicking the habit/semiconductor lasers without isolators. *Opt. Express* **28**, 36466 (2020).
9. Oldenbeuving, R. M. *et al.* 25 kHz narrow spectral bandwidth of a wavelength tunable diode laser with a short waveguide-based external cavity. *Laser Phys. Lett.* **10**, 015804 (2012).
